# Trismus Nascentium

**Published:** 1867-04

**Authors:** Jno. M. Langhorn

**Affiliations:** Uniontown, Ala.


### ARTICLE III.

*Trismus Nascentium.* By JNO. M. LANGHORN, M. D., of Uniontown, Ala.

DR. J. M. JOHNSON'S remarks, introductory to an article on the diet of infants, by Dr. Cummings, that "were mothers to hold their infants by the heels occasionally, in imitation of the position occupied by the fœtus for some months prior to its birth, many cases of Trismus Nascentium might be prevented," suggests to me the enquiry, whether, in the altered circumstances of the case after birth, there is not ample compensation for the lost effect of gravity merely, in the fœtal state, to be found in the stimulating effect of oxygen entering the lungs of the newly born infant, notwithstanding the fact, which is unquestionable, that the more general distribution of the blood, especially in the direction of the lungs, does abstract the stimulus of mechanical pressure from the brain. But is not this more likely to produce syncope than trismus? Amongst the various opinions concerning the cause of trismus nascentium, whether from the shock caused by violent protracted pressure during parturition, or from the dorsal decubitus of the infant, practiced by many mothers, (negroes especially) supposed to produce cerebral irritation from the overlapping of the parietal bones, or the congenital obstruction of the ductus communis, resulting in the jaundiced condition which is commonly met with. I am inclined to the opinion, that none of these explain the true cause of the disease; as we have in puerperal mania and phrenitis and meningetis, examples of kindred affections to the former, whilst we have in hepatitis an example of the latter. But in none of these affections do we witness the symptoms of trismus nascentium. But we have a source as constant as the multiplied millions that are ushered into life, whence may legitimately arise the disease in question. I refer to the umbilicus. Here is, at birth, an aperture for the accommodation of the fœtal vessels, which must be closed by the contraction of its marginal bandage to a focus; more or less constrictory force is engaged, according to the presence or absence of inflammation during the process of closing and cicatrization; the navel is always more sensitive even in health than the surrounding parts. Now, we may find here circumstances singularly opposite to the cause of traumatic affections. A mechanical cause operating upon a highly sensitive aponeurotic (in its literal sense) center, similar in its effect and operation to that of a foreign body, in contact with a nerve, invariably associated with symptoms of inflammation of the umbilicus at first, which may disappear for the most part upon cicatrization, to be propagated to the peritoneum, and thence to intestines and liver, resulting in grave disturbance of the functions of the latter.

We find trismus nascentium to occur, and always about the 9th day—on that of the culmination of acute diseases—and with such uniformity in this particular, as to be commonly known as 9th day fits.

We believe, then, that the disease invariably proceeds from this cause, and that it is associated with grave peritoneal inflammation, transmitted through the medium of inflammation of the fœtal vessels, and subsequently reflected to the whole abdominal viscera, and have, accordingly, addressed ourselves to the treatment of it, with results which, according to all the light which we have upon the subject, a parallel in success.

We sometimes have found an elevated and inflamed and hardened ring around the now obliterated aperture which we have nicked in several places with the lancet, and at the same time we apply spirits of turpentine to promote suppuration of the part, whilst we cover the whole abdomen with a poultice smeared over thickly with turpentine and lard, or olive oil, in equal parts, which we keep constantly applied, and removed frequently, and give the following:

℞—Calomel, grs. viij.
Ipecac, grs. iij.
Prepared Chalk, grs. xxxvj.
Ext. of Hyoscyamus, grs. viij.
Fiat Chact. no. xij.—S. one every 3 hours.

And at the same time, we give Tinct. Cannabis Imdica, 8 to 12 drops every half hour—ascending or descending in the dose as the case may require. Under this plan of treatment, we have cured one half of the cases treated, when we have been notified of the first appearance of the symptoms; and believe that were our efforts properly seconded, even a larger proportion might be saved.

